# Population-based meta-analysis of chloroquine: informing chloroquine pharmacokinetics in COVID-19 patients

**DOI:** 10.1007/s00228-020-03032-6

**Published:** 2020-11-13

**Authors:** Xueting Yao, Xiaoyu Yan, Xiaohan Wang, Ting Cai, Shun Zhang, Cheng Cui, Xiaoxu Wang, Zhe Hou, Qi Liu, Haiyan Li, Jing Lin, Zi Xiong, Dongyang Liu

**Affiliations:** 1 Drug Clinical Trial Center, Peking University Third Hospital, Beijing, 100191 China; 2 School of Pharmacy, Faculty of Medicine, The Chinese University of Hong Kong, Shatin, New Territories, Hong Kong Special Administrative Region, 999077 China; 3 School of Basic Medicine and Clinical Pharmacy, China Pharmaceutical University, Nanjing, 211198 China; 4 Key Laboratory of Diagnosis and Treatment of Digestive System Tumors of Zhejiang Province, HwaMei Hospital, University of Chinese Academy of Sciences (Ningbo No.2 Hospital), Ningbo, 315010 China; 5 Department of Orthopedics, Peking University Third Hospital, Beijing, 100191 China; 6 Department of Cardiology and Institute of Vascular Medicine, Peking University Third Hospital, Beijing, 100191 China

**Keywords:** Chloroquine, Population pharmacokinetics, Coronavirus disease 2019

## Abstract

**Aims:**

Chloroquine (CQ) has been repurposed to treat coronavirus disease 2019 (COVID-19). Understanding the pharmacokinetics (PK) in COVID-19 patients is essential to study its exposure–efficacy/safety relationship and provide a basis for a possible dosing regimen optimization.

**Subject and methods:**

In this study, we used a population-based meta-analysis approach to develop a population PK model to characterize the CQ PK in COVID-19 patients. An open-label, single-center study (ethical review approval number: PJ-NBEY-KY-2020-063-01) was conducted to assess the safety, efficacy, and pharmacokinetics of CQ in patients with COVID-19. The sparse PK data from 50 COVID-19 patients, receiving 500 mg CQ phosphate twice daily for 7 days, were combined with additional CQ PK data from 18 publications.

**Results:**

A two-compartment model with first-order oral absorption and first-order elimination and an absorption lag best described the data. Absorption rate (ka) was estimated to be 0.559 h^−1^, and a lag time of absorption (ALAG) was estimated to be 0.149 h. Apparent clearance (CL/F) and apparent central volume of distribution (V2/F) was 33.3 l/h and 3630 l. Apparent distribution clearance (Q/F) and volume of distribution of peripheral compartment (Q3/F) were 58.7 l/h and 5120 l. The simulated CQ concentration under five dosing regimens of CQ phosphate were within the safety margin (400 ng/ml).

**Conclusion:**

Model-based simulation using PK parameters from the COVID-19 patients shows that the concentrations under the currently recommended dosing regimen are below the safety margin for side-effects, which suggests that these dosing regimens are generally safe. The derived population PK model should allow for the assessment of pharmacokinetics–pharmacodynamics (PK-PD) relationships for CQ when given alone or in combination with other agents to treat COVID-19.

**Supplementary Information:**

The online version contains supplementary material available at 10.1007/s00228-020-03032-6.

## Introduction

The outbreak of coronavirus disease 2019 (COVID-19) has quickly become a global pandemic since December 2019. Although no treatment has demonstrated clinical efficacy against COVID-19, various drugs are being repositioned to treat COVID-19 and are being tested clinically. Chloroquine (CQ) phosphate has been shown to effectively suppress SARS-CoV-2 in vitro assay [1, 2]. Preliminary data from clinical trials also demonstrate its beneficial effect such as reducing the deterioration of pneumonia, improving the lung-imaging results, decreasing the viral load, and shortening the disease duration [3]. This drug is recommended in the guidelines for the prevention, diagnosis, and treatment of pneumonia caused by COVID-19, issued by the National Health Commission of the People’s Republic of China [4]. The US Food and Drug Administration has also issued an emergency use authorization (EUA) to permit the emergency use of chloroquine phosphate in patients with COVID-19 under certain conditions [5].

Chloroquine has been shown to be widely distributed in tissues and organs, where apparent distribution volume was up to 13,000–65,000 l (about 200 l/kg in whole blood and 800 l/kg in plasma) [6]. About 30%–50% of the CQ was metabolized in the liver by CYP2C8, CYP3A4, and CYP2D6, where CYP2C8 contributed about 60% and CYP3A4 contributed 25% of metabolism [7]. The accumulation of CQ in organs and blood cells resulted in its relatively slow clearance, and a long half-life of 20–60 days [6]. So far, no PK data of CQ in COVID-19 patients has been reported.

Chloroquine phosphate has been used for the treatment of malaria and autoimmune diseases for over 70 years. It is also used for prophylaxis of malaria for people returning from malaria-endemic geographic areas [8]. According to the prescribing information, the dosage on the first day is not to exceed 1500 mg, followed by a daily maintenance dose not to exceed 1000 mg. The major safety concern involves QT prolongation, ventricular tachycardia, and retinopathy [9]. The cardiotoxicity after CQ administration included hypotension, QT interval prolongation, cardiomyopathy, arrhythmia, and ventricular tachycardia. After receiving 600 mg CQ, adult volunteers had a mean 16 ms (95% confidence interval 9–23) prolongation of the Bazett corrected QT interval [10]. The mean CQ IC_50_ value for hERG channel inhibition in *Xenopus* oocytes has been mesasured at 8.4 μmol/l (2700 ng/ml) [11], which suggested that only very high concentrations in vivo might cause clinically significant QT prolongation.

The effect of chloroquine in COVID-19 patients is still waiting to be seen. Understanding its pharmacokinetics is important in order to study its exposure–efficacy/safety relationship and provide a basis for its dosing regimen. The purpose of this study was to develop a population PK model of CQ based on the literature data as well as the data from COVID-19 patients.

## Methods

### Clinical pharmacokinetic study of COVID-19 patients

An open-label, single-center study (ethical review approval number: PJ-NBEY-KY-2020-063-01) was conducted to assess the safety, efficacy, and pharmacokinetics of CQ in patients with COVID-19. The study was approved by the Ethics Committee of Ningbo Hwamei Hospital, University of Chinese Academy of Sciences (Ningbo, China), and was performed in accordance with the Declaration of Helsinki.

Patients who met the inclusion criteria but did not have any of the exclusion criteria were included in this study. Inclusion criteria included: 

1) Being aged 18 years old or older

2) Having been diagnosed with COVID-19 and meeting all of the following criteria: (A) had an epidemiological history, (B) had clinical manifestations (met any two of the following — fever; normal or decreased white blood cell count or lymphopenia in the early stage of onset; and chest radiology in the early stage showing multiple small patchy shadowing and interstitial changes, which is especially significant in periphery pulmonary (furthermore, this develops into bilateral multiple ground-glass opacity and infiltrating shadowing. Pulmonary consolidation occurs in severe cases. Pleural effusion is rare), and (C) suspected cases who had one of the following etiological evidence and had consequently been confirmed as COVID-19: respiratory or blood specimens testing positive for novel coronavirus nucleic acid by real-time fluorescent RT-PCR; respiratory or blood specimen virus gene sequencing had shown them to be highly homologous with the known novel coronavirus.

 These patients received CQ phosphate administration unless they had one or more of the following exclusion criteria: 1) female patients in pregnancy, 2) patients with a clear history of allergies to chloroquine, 3) patients suffering from diseases of the blood system, 4) patients suffering from chronic liver or kidney diseases and reaching the terminal stage, 5) patients suffering from arrhythmia or chronic heart disease patients, 6) patients with known retinal diseases or hearing loss, 7) patients with known mental illness, and 8) patients who have to use digitalis drugs for existing underlying diseases.

All enrolled subjects signed the Informed Consent Form (ICF) before the study was conducted. Subjects received 500 mg CQ phosphate (300 mg CQ) twice a day for 7 days continuously. Blood samples of 4 ml on days 1, 3, 7, and 14 were collected prior to dose administration. Additional sparse blood samples were collected. Anticoagulation of ethylene diamine tetraacetic acid (EDTA) was used to separate plasma after centrifugation for 5 min at 3000 rpm. The collected plasma samples were stored at −80 °C before analysis. The plasma concentrations of CQ were determined using a validated high-performance liquid chromatography-tandem mass spectrometry (HPLC-MS/MS) method (see details in supplementary file). The lower limit of quantitation (LLOQ) was 2.00 ng/ml. The accuracy was within the range of ± 15%, and the precision was less than 15%.

All subjects receiving at least one dose were incorporated into the analysis. The patient demographics, baseline characteristics (including laboratory examination), and drug combination information were also collected and included in the analysis.

### Literature data collection

All published literature on clinical PK of CQ was collected from PubMed and Embase databases. The keywords used for searching were: “Clinical Pharmacokinetic and Chloroquine”. Publications from January 1, 1940, to February 29, 2020, were reviewed. Inclusion criteria of publications for PK model development were: 1) the study drug was chloroquine phosphate or chloroquine, 2) human, as the research subjects had participated in clinical trials, 3) the literature reported the plasma CQ concentration-time profiles. Exclusion criteria were: 2) literature did not clearly describe the dosage and the demographics of subjects, 2) the reported CQ concentration-time profiles were too vague for data extraction, 3) literature reported only serum or blood drug concentration instead of plasma concentration.

Aggregate (mean) plasma CQ PK profiles of identified publications were extracted together with the dosing information. The number of subjects that contributed data to each aggregate profile was included as a variable in the analysis dataset. An indicator variable was also created to appoint the PK profiles as either aggregate or individual PK data to allow for separately estimating their residual errors.

### Population PK model development

#### Structural PK model

**Fig. 1:**
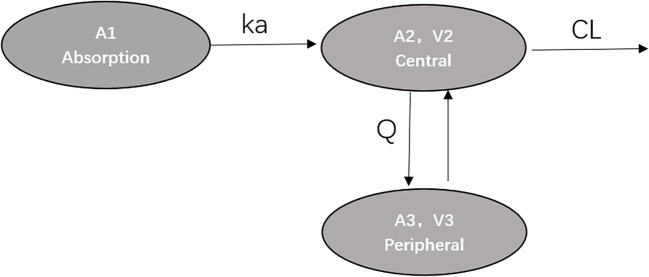
Chloroquine population pharmacokinetic model structure

A two-compartment model with first-order oral elimination and absorption with an absorption lag was developed to describe the plasma concentration-time course of CQ (Fig. 1). The same model structure has been used in the literature to describe the PK of CQ [12–14]. The model parameters estimated in the structural PK model included first-order oral absorption (ka), apparent clearance (CL/F), apparent inter-compartmental clearance (Q/F), volume of distribution for the central compartment (V2/F), volume of distribution for the peripheral compartment(V3/F), and lag-time (ALAG). An exponential error model was used to characterize inter-individual variability for each parameter, where possible, assuming log-normal parameter distributions. Residual variability (σ^2^) for the plasma concentration data was evaluated using separate proportional error models for aggregate data from literature and individual COVID-19 patients. For aggregate profile, the residual error was weighted by the inverse of the square root of the number of individuals that contributed data to an aggregate plasma PK profile [15]. In addition to population model-based meta-analysis, the current suggested methodology for stabilizing pharmacokinetic model when analysis the sparse data is to use the $PRIOR functionality in NONMEM, which can make the model run successfully by using a priori information [16, 17]. This methodology was also conducted and compared. The population pharmacokinetic literature about chloroquine on PubMed was searched, and the information then collected and summarized. The meta-analysis of previous population pharmacokinetic studies was conducted, and the summarization of the PK parameters is provided in Table S2 in the supplementary file. The $PRIOR function of the NONMEM software was then used to rerun the model.

#### Covariate analysis

The main purpose of covariate analysis was to investigate the effects of covariates on CL/F and V2/F. Since many covariates were not reported in the literature, the aggregate data from literature and clinical PK data was only used to establish the base model. For covariate analysis, the model parameters were fixed to estimated values from the base model except for parameters of random effects for CL/F and V2/F. Then, this model was used to investigate the influence of covariates based on only the PK and covariates data from COVID-19 patients. The demographic data included race, age, and body weight. Baseline laboratory tests of COVID-19 patients were also analyzed, which included alanine aminotransferase (ALT), albumin (ALB), aspartate transaminase (AST), serum creatinine (SCr), direct bilirubin (DBIL), total bilirubin (TBIL), body temperature baseline (TEMB), highest body temperature in hospital (TEMP), white blood cell count (WBC), blood platelet count (PLT), hemoglobin (HGB), D-dimer, hematocrit (HCT), and creatinine clearance (CrCL). Missing covariates that were less than 5% of total covariates data were imputed by the median values, while missing covariates that were equal to or greater than 5% of total covariates data were not included in analysis. The covariates that were evaluated on PK model parameters are summarized in Table S1 in the supplementary file. The covariates were incorporated into the base model using the step-wise screening approach, which was implemented manually through forward selection and backward elimination. Power and proportional covariate models of covariate effects on PK parameters were tested for continuous variables and categorical variables respectively. Potential covariates were entered one by one into the population PK model. When the objective function value (OFV) was reduced by more than 3.84 with degrees of freedom (df) equal to 1 (*p* < 0.05), the covariate was kept in the model. After the development of a full multivariable model, it was checked by subtracting each covariate individually using backward elimination. Where OFV was increased by more than 6.63 (*p* < 0.01, df = 1), the subtracted covariate was put back into the model. Allometric scaling models using body weight normalized PK parameters to size were also evaluated [18].

#### Model evaluation and validation

The final population PK model was assessed using the goodness-of-fit (GOF) plots, which included the dependent variable (DV) versus individual prediction (IPRED) or population prediction (PRED), conditional weighted residuals (CWRES) versus PRED, and CWRES versus time. Prediction-corrected visual predictive check (pcVPC) [19] was also produced, which was conducted based on simulations of 1000 replicates .

#### Model simulation

The simulation of PK profile following various CQ phosphate dosing regimens was conducted using the individual PK parameter values from COVID-19 patients. Different dose regimens of CQ phosphate were under consideration including the standard treatment for antimalarial, the efficacious dose clinically observed [3], and recommended dose according to our previous work on the CQ physiologically based pharmacokinetic (PBPK) study. Five dosing regimens of CQ phosphate (Table 1) were proposed for the treatment of COVID-19 patients. The plasma concentration of CQ under five dosing regimens were simulated using the final population PK model. The individual PK parameters of the 50 COVID-19 patients were used to conduct simulations under each dosing scenario. The 5th, 50th, and 95th percentiles of simulated plasma CQ concentrations were plotted over time. Meanwhile, a safety margin was proposed. Chronically treated patients with serum CQ concentration below 1.3 μmol/l (416 ng/ml) demonstrated no side-effects, but 80% of patients experienced side-effects when their serum concentration was above 2.5 μmol/l (800 ng/ml) [15, 19]. Assuming plasma concentrations are equivalent to serum concentrations, a plasma concentration of 400 ng/ml was selected as a safety limit, and the warning limit was set at a maximum concentration of 800 ng/ml.

**Table 1:** Chloroquine phosphate dosing regimen simulated in Fig. 5

No.	Rational	Dosing regimen for chloroquine phosphate	Reference
1	The standard dosing for the antimalarial treatment	1000 mg on day 1, 500 mg after 6 h, followed by 500 mg QD for day 2 and 3	[9]
2	The recommended dosing for the treatment of COVID-19 patients (body weight > 50 kg) from Diagnosis and Treatment for COVID-19 (7th version) issued by the China National Health Commission	500 mg BID for 7 days	[4]
3	The recommended dosage for acute COVID-19 patients	750 mg on day 1, 500 mg after 6 h, followed by 500 mg QD till day 5	[25]
4	The recommended dosage for moderate COVID-19 patients	750 mg on day 1, 500 mg after 6 h, followed by 500 mg BID on day 2 and 3, 250 mg BID on day 4 and 5	[25]
5	The recommended dosage for vulnerable patients who may need a reduced dose	250 mg BID for 5 days	[25]

Note: This table is attached to Fig. 5 to clarify the dosing regimen simulated in Fig. 5

#### Software and platform used

Dataset arrangement and exploratory data analysis were performed using R (version 3.5.3, https://www.r-project.org/) and RStudio (version 1.1.453, https://rstudio.com/). A nonlinear mixed-effects model was implemented in NONMEM (version 7.3, Icon Development Solutions, Ellicott City, MD, USA) interfaced by Pirana (version 2.8) and Perl speaks NONMEM (PsN) (version 3.6.2) toolkit [20]. Model-based simulations were conducted using the R mrgsolve package. CQ mean concentration-time profiles were extracted using Plot Digitizer (GetData, Version 2.26).

## Results

### Clinical PK study

A total of 50 COVID-19 patients (19 males and 31 females) who received 500 mg CQ phosphate (300 mg CQ) for at least one dose were included in analysis. Demographic and baseline characteristics of COVID-19 patients are summarized in Table 2. A total of 315 PK observations from patients were managed in the dataset, and 15 observed concentrations were below the limit of quantification. The trough CQ concentrations (standard deviation) at day 1, day 3, day 7, and day 14 were 117.3 (50.7), 291.5 (45.5), 280.5 (27.5), and 199.3 (65.2) ng/ml respectively.

**Table 2:** Demographic and baseline characteristics (mean ± SD) of COVID-19 patients who participated in the clinical trial

Demographic and baseline	Subjects (*n* = 50)
Age (year)	53.46 ± 15.46
Sex (*n*)	Male = 19, female = 31
Body-mass index (kg/m^2^)	23.97 ± 3.40
Height (m)	1.63 ± 0.08
Weight (kg)	64.17 ± 10.91
Body temperature baseline (°C)	37.14 ± 0.64
White blood cell count (×10^9^/l)	4.95 ± 1.76
Blood platelet count (×10^9^/l)	201.76 ± 81.11
Hemoglobin (g/l)	132.94 ± 14.37
Alanine aminotransferase (IU/l)	25.55 ± 18.93
Aspartate transaminase (IU/l)	25.54 ± 12.48
Neutrophil (×10^9^/l)	3.37 ± 1.55
Lymphocytes (×10^9^/l)	1.12 ± 0.53
Monocytes (×10^9^/l)	0.38 ± 0.17
Hematocrit (%)	40.27 ± 3.83
D-dimer (ng/ml)	166.44 ± 173.39
Prothrombin time (s)	12.29 ± 1.12
Total bilirubin (μmol/l)	10.93 ± 6.03
Direct bilirubin (μmol/l)	3.77 ± 2.18
Total protein (g/l)	70.27 ± 5.32
Serum creatinine (mg/dl)	0.70 ± 0.21
Creatinine clearance^*^ (ml/min)	106.19 ± 33.88
Total cholesterol (mmol/l)	4.04 ± 0.79
Triglyceride (mmol/l)	1.51 ± 1.01

*Creatinine clearance was calculated using Cockcroft–Gault equation

For men, $$ \mathrm{Creatinine}\ \mathrm{clearance}=\frac{\left(\ 140- age\right)\times weight}{72\times serum\ creatinine} $$

For women, $$ \mathrm{Creatinine}\ \mathrm{clearance}=0.85\times \frac{\left(140- age\right)\times weight}{72\times serum\ creatinine} $$

### Summary of the literature data

Extracted from literature, additional CQ PK data were obtained from 18 published literature reports (Table 3). One study on healthy children and Kwashiorkor children, one study on pregnant women, one study on patients with Acute vivax malaria, and nine studies on healthy subjects were included in the meta-analysis. In addition, 18 CQ mean concentration-time profiles were extracted. The dose of CQ ranged from 50 mg to 600 mg, and contained a single-dose administration and multiple-dose administrations (e.g., once a day or once a week). A total of 220 PK data points from the literature were included in the dataset.

**Table 3:** Summary of published literature reports on chloroquine pharmacokinetics

Study number	Reference	Studied population	Chloroquine doses	Number of treatment periods	Number of individuals generated	Data points
1	Walker O et al., 1987	Children (*n* = 6)	10 mg/kg	1 (single oral dose)	1	12
		Kwashiorkor Children (n = 5)	10 mg/kg	1 (single oral dose)	1	11
2	Harin A et al., 2010	Pregnant (*n* = 30)	450 mg	1 (oral daily dose for 3 days)	1	6
		Healthy (*n* = 30)	450 mg	1 (oral daily dose for 3 days)	1	6
3	Gustafsson LL et al., 1983	Healthy (*n* = 11)	300 mg	1 (single oral dose)	1	18
4	Neuvonen PJ et al., 2009	Healthy (*n* = 6)	310 mg	1 (single oral dose)	1	9
5	Pukrittayakame S et al., 2014	Healthy (*n* = 16)	600 mg	1 (single oral dose)	1	19
6	Walker O et al., 1987	Healthy (*n* = 8)	600 mg	1 (single oral dose)	1	12
7	de Vries PJ et al., 1994	Healthy (*n* = 19)	600 mg	1 (single oral dose)	1	17
8	Oosterhuis B et al., 1981	Healthy (*n* = 3)	600 then 300 mg	1 (600–600-300 mg at t = 0, 24 and 48 h)	1	17
		Healthy (*n* = 2)	600 then 300 mg	1 (600–600-300 mg at t = 0, 24 and 48 h)	1	17
9	Onyeji CO et al. 2001	Healthy (*n* = 18)	600 mg	1 (single oral dose)	1	9
		Healthy (*n * = 18)	600 mg	1 (single oral dose)	1	9
10	Daher A et al., 2019	Acute vivax malaria (*n* = 58)	600 then 450 mg	1 (600 mg on day 1, and 450 mg on days 2 and 3)	1	6
11	Onyeji CO et al., 1993	Healthy (*n *= 6)	300 mg	1 (single oral dose)	1	9
12	Wetsteyn JCFM et al., 1995	Healthy (n = 5)	300 mg	1 (3 weeks: once weekly 300 mg)	1	13
		Healthy (*n* = 4)	200 mg	1 (3 weeks: twice weekly 200 mg)	1	15
		Healthy (*n* = 5)	50 mg	1 (3 weeks: once daily 50 mg)	1	15
Total					18	220

### Model development and evaluation

A two-compartment model with first-order oral absorption and elimination (Fig. 1) best described the CQ plasma concentration. The CL/F of CQ was estimated to be 33.3 l/h, absorption rate ka was estimated to be 0.559 h^−1^, and ALAG was estimated to be 0.149 h. The volume of distribution in central and peripheral compartments was 3630 l and 5120l respectively. Inter-compartment clearance Q/F was estimated to be 58.7 l/h. The inter-individual variability (IIV) was estimated for ka, CL/F, V2/F, V3/F, and Q/F. Due to the high inter-individual variability of COVID-19 patients and the inter-studies variability, the IIV for PK parameters were expressed as CV% and ranged from 44.8% to 67.7%, except for IIV of ka (110.5%). The RSE% of fixed- and random-effect parameter estimates were less than 40%. The results for $PRIOR functionality in NONMEM were shown in the following Table S3 and Fig. S1 in the supplementary file. As a result, the parameters estimated by the model are close to the results obtained by our previous method, but the RSE% values of ka and ALAG1 were very large and were not met the acceptable criteria.

During covariate analysis, no demographic or laboratory examination baseline variates were identified to have a significant effect on CQ PK. Since body weight has been identified as a significant covariate for CQPK [12], positive relationship of body weight on CL/F and V2/F was integrated into the PK model, and the decrease objective function value was 14.1 (corresponding to a *p* value of 0.01). The final model included body weight and used a fixed allometric coefficient of 0.75 for CL/F and 1 for V2/F [12]. The final model parameters estimates are summarized in Table 4. The condition number of the final model was 52.8.

**Table 4:** Model parameter estimation for meta-analysis and observed data

Parameter	Final estimate	% RSE	Bootstrap median (95 CI%)
CL/F (l/h)	33.3	8.00	33.7 (29.2, 38.6)
V2/F (l)	3630	13.3	3598 (2521, 4532)
Q/F (l/h)	58.7	15.4	56.0 (43.7, 72.0)
V3/F (l)	5120	11.8	5044 (4274, 6089)
ka (h^−1^)	0.559	20.2	0.607 (0.370, 1.12)
ALAG1 (h)	0.149	20.4	0.149 (0.130, 0.320)
Weight on CL/F	0.75 FIX	/	/
Weight on V2/F	1 FIX	/	/
ω^2^ for CL/F	48.8	8.50	47.4 (35.8, 61.3)
ω^2^ for V2/F	67.7	14.0	67.6 (49.8, 88.5)
ω^2^ for Q/F	48.4	26.1	46.8 (21.8, 63.8)
ω^2^ for V3/F	48.2	18.4	48.1 (27.8, 63.8)
ω^2^ for ka	111	40.5	106 (54.8, 139)
σ^2^ for individual data	24.7	4.20	24.7 (19.4, 28.4)
σ^2^ for aggregate data	59.7	4.60	58.6 (48.1, 72.5)

IIVs (ω^2^) and residual errors (σ^2^) are expressed as coefficients of variation (%)

**Fig. 2:**
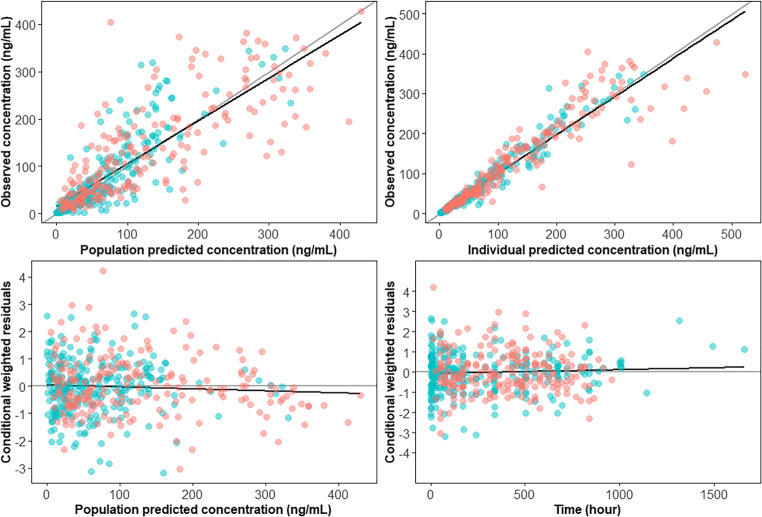
Goodness of fit plots for the final model. The *blue dots* represent the aggregate data. The* red dots* represent the data from individual COVID-19 patients. The *black solid line* represents a linear smooth line. The *gray diagonal lines* (*top panels*) and *horizontal lines* (*bottom panels*) are the lines of identity and zero lines respectively

**Fig. 3:**
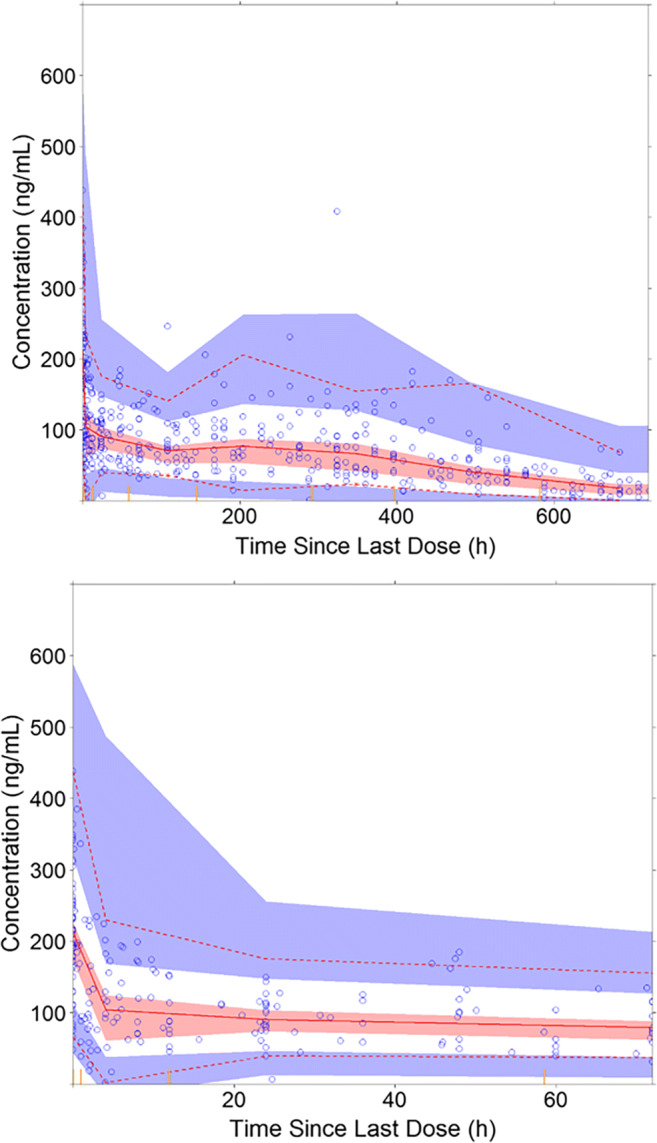
Prediction-corrected VPC (pcVPC) of the final model. The *top panel* represents pcVPC plot of 0–720 h; the *bottom panel* represents pcVPC plot of 0–72 h. The *blue circles* represent chloroquine concentration data. The *upper red dotted line*, the *middle red solid line*, and the *lower red dotted line* represent 95%, 50%, and 5% quantiles of observed data respectively. The *shaded area* represents a 90% confidence interval for 95%, 50%, and 5% quantiles of observed data

**Fig. 4:**
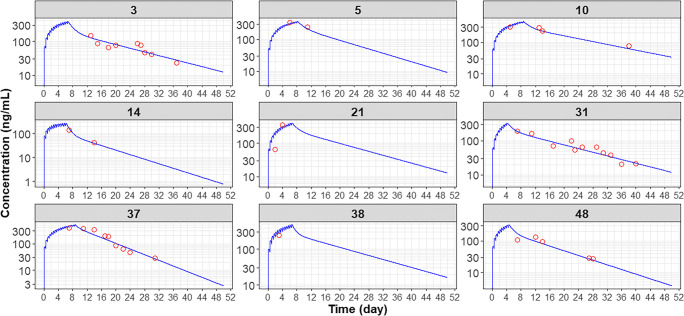
Observed vs individual predicted plasma concentration for selected subjects

As depicted in Fig. 2, GOF plots of the population- and individual- predicted plasma CQ concentrations versus observed concentrations showed no major bias. The conditional weighted residuals (CWRES) versus population-predicted concentrations or versus time after dose showed that most of CWRES were within the range of (−2, 2). The pcVPC revealed that there was reasonable agreement between the observed and model-predicted concentrations over time (Fig. 3). Figure 4 shows the time-course of observed versus individual predicted plasma CQ concentrations from nine representative patients. Overall, these model diagnostics suggest that the final population PK model adequately describes the CQ plasma data.

### Model simulation

**Fig. 5:**
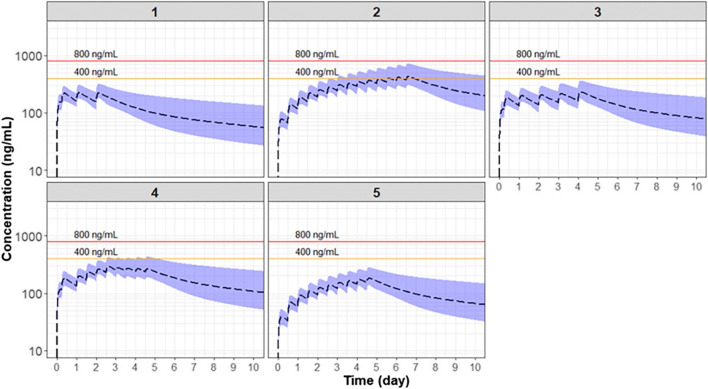
Simulations of chloroquine PK under various dosing regimens. The *black dashed line* represents the 50th percentile and the *shaded area* represents the range between 5th and 95th percentile. The *solid line* represents the safety concentration threshold of 400 mg/ml and 800 mg/ml

The simulated CQ median concentrations with 90% predictive interval under five dosing regimens were shown in Fig. 5. The simulation results show that the predicted population mean and individual C_max_ of regimen 1 (conventional antimalarial treatment), regimen 2, and regimen 5 were below 400 ng/ml. Under regimen 2, the model simulated population median C_max_ of plasma exceeded 400 ng/ml, but was below 800 ng/ml. The simulated population median C_max_ of regimen 3 was below 400 ng/ml, while 95th percentile of simulated plasma CQ concentrations slightly exceeded 400 ng/ml, but was below 800 ng/ml.

## Discussion

Characterization of the PK of CQ in COVID-19 patients is essential to evaluate the exposure–safety/efficacy relationship of CQ and subsequent dosing regimen optimization. However, traditional PK analysis is not possible with the current study because only sparse PK samples are available. Thus, we developed a population-based PK model based on both the meta-individual from the literature and the individual patient in the study. Such a method has been applied in the literature to address critical gaps in experimental PK data, especially when the PK of the drug has been extensively studied [21, 22]. The developed PK model allows us to obtain individual PK parameters for each patient and predict their exposure metrics in the trial.

A two-compartment model with first-order absorption best describes the time course of CQ in patients. This is consistent with other publications on PK modeling of CQ for various patient populations including children, pregnant women, healthy adults, and malaria patients [12–14, 23]. The estimated fixed-effect PK parameter generally agrees with those from other publications. Body weight has been included as a significant covariate for CQ clearance and volume of distribution, because the previous publication has identified body weight as a significant covariate for CQ PK [12]. We think that using the functionality in NONMEM, the results were unacceptable for the following reasons: 1).the number of literature studies (*n* = 5) that met the requirements was small, which cannot provide sufficient prior information, 2) the subject type in the literature was inconsistent, including healthy subjects, infants, and pregnant women, and 3) there was a 10-fold difference on the same PK parameter among the five studies, meaning that there was a large inconsistency among the prior information. Therefore, we think that our current approach was more appropriate for establishing a population pharmacokinetic model of chloroquine in COVID-19 patients.

The safety of CQ has been always a major concern in clinical treatment, especially at high drug concentrations. The chronically treated patients with serum CQ concentration below 1.3 μmol/l (416 ng/ml) demonstrated no side-effects, but 80% of patients experienced side-effects when their serum concentration was above 2.5 μmol/l (800 ng/ml) [24, 25]. A retrospective study found that ingestion of more than 5 g CQ caused severe CQ poisoning and fatal outcome, where CQ blood concentration was more than 25 μmol/l, corresponding to the plasma CQ concentration of 3.5 μmol/l (1120 ng/ml) [26]. For a conservative safety margin, a safe plasma concentration of 400 ng/ml and a vigilant plasma concentration of 800 ng/ml was selected.

Model-based simulations were conducted to evaluate the PK profile under various dosing regimens (Fig. 5). The results are generally consistent with the previous prediction using the PBPK model approach for the same dosing regimens [27]. The simulation results from regimens 1 and 3 show that the loading dose is effective in achieving a faster onset of drug exposure. Also, the concentrations at various dosing regimens are below the 800 ng/ml threshold, indicating that these regimens are generally safe. Based on the simulation results (Fig. 5), the maximum CQ plasma concentration in all designed dosing regimens was below the vigilant threshold, except for regimen 2. The rest of the regimens yielded concentrations below 400 ng/ml.

The therapeutic concentration of CQ in COVID-19 patients has not yet been established. The reported in-vitro EC_50_ of chloroquine inhibiting SARS-CoV-2 virus ranges from 1.13 to 23.9 μmol/l (corresponding to 362 to 7646 ng/ml) [1, 2, 28]. This concentration represents the free drug concentration in the medium. Based on Fig. 5, the plasma CQ concentration appears to be lower than the reported in-vitro EC_50_ at these dosing regimens. Considering about 55% of the chloroquine in the plasma is bound to protein [6], the gap between free drug concentration in plasma and in-vitro EC_50_ is even bigger. However, it should be noted that the in-vitro EC_50_ should be compared to the drug concentration in the interstitial fluid or intracellular fluid in the lungs, which is not available. However, it is known that chloroquine is heavily accumulated in the lungs [29]. Furthermore, the mechanism of chloroquine against COVID-19 is still unclear. Chloroquine may achieve the therapeutic effect via pathways other than directly inhibiting the virus. Savarino et al. hypothesized that CQ might inhibit the production of pro-inflammatory cytokines (such as interleukin-6), thereby blocking the pathway that leads to acute respiratory distress syndrome [30]. Nevertheless, the in-vivo effective concentration of CQ remains to be defined. Future PK/PD analysis based on various biomarkers and clinical endpoints is warranted.

The current analysis is not without limitation. For instance, because the number of patients, concentrations, and covariate data in COVID-19 patients were limited, it might be difficult to identify significant covariate effects, if there is any. The gene polymorphism of CYP2C8, one of the main metabolic enzymes of CQ, was also not evaluated because the data was not available. In summary, a population PK model for CQ was developed using a population-based meta-analysis approach. The model was constructed using combined PK data from the meta-individual in the literature and individual COVID-19 patients, which provides confidence that this model can reasonably characterize CQ PK in the patient population. The derived population PK model should allow for the assessment of PK-PD relationships for CQ when given alone or in combination with other agents to treat COVID-19.

## Electronic supplementary material

ESM 1 (DOCX 452 kb)

## Data Availability

All data used in this study were reported in the manuscript.
