# Supplementary material for: Population-based meta-analysis of chloroquine: informing chloroquine pharmacokinetics in COVID-19 patients
Source: Eur J Clin Pharmacol. 2020 Nov 13;77(4):583–93. doi: 10.1007/s00228-020-03032-6 (PMC7665884; doi:10.1007/s00228-020-03032-6)

***NONMEN code for final model：***

;; 1. Based on: run2

;; 2. Description: CL+V2-WT

;; x1. Author: user

$PROBLEM TWO COMPARTMENTAL MODEL

$INPUT ID NUM TIME DV CMT AMT MDV EVID ADDL II IND DOSE WEIGHT TAD

$DATA 0416NM_V2.csv IGNORE=@

$SUBROUTINES ADVAN4 TRANS4

$PK

CL = THETA(1)*EXP(ETA(1))*(WEIGHT/65.50)**THETA(7) ;CLEARANCE

V2 = THETA(2)*EXP(ETA(2))*(WEIGHT/65.50)**THETA(8) ;VOLUME OF CENTRAL COMPARTMENT

Q = THETA(3)*EXP(ETA(3)) ;INTERCOMPARMENTAL CLEARANCE

V3 = THETA(4)*EXP(ETA(4)) ;VOLUME OF PERIPHERAL COMPARMENT

K23=Q/V2 ;Distribution rate constant

K32=Q/V3 ;Distribution rate constant

KA=THETA(5)*EXP(ETA(5))

S2=V2/1000

ALAG1=THETA(6)

$ERROR

IF (CMT.EQ.2) THEN

IPRED=F

;IPRED=A(2)/V2*1000

W=1/(SQRT(NUM))

Y = IPRED + IPRED*EPS(1)*IND+IPRED*EPS(2)*(1-IND)*W

ENDIF

IRES=DV-IPRED

$THETA

(0,28.80) ; THETA1 CL

(0,2942.97) ; THETA2 V2

(0,51.89) ; THETA3 Q

(0,4964.10) ; THETA4 V3

(0,0.72) ; THETA5 KA

(0,0.11) ;THETA6 ALAG1

0.75 FIX ; THETA7 CL-WT

1.00 FIX ; THETA8 V2-WT

$OMEGA

0.20 ; ETA1 ON CL

0.20 ; ETA2 ON VC

0.10 ; ETA3 ON Q

0.20 ; ETA4 ON V2

0.42 ; ETA4 ON KA

$SIGMA

0.54

0.52

$EST MAXEVAL=9999 METHOD=1 PRINT=1 SIGDIGITS=2 INTERACTION NOABORT MSFO=RUN9.MSF

$COV PRINT=E MATRIX=S

$TABLE ID TIME DV AMT MDV KA CL V2 V3 Q CMT WEIGHT TAD CWRES IPRED IRES NOPRINT ONEHEADER FORMAT=s1PE17.10 FILE=mytab9

**HPLC-MS/MS method validation of chloroquine determination in human plasma**

A high-performance liquid chromatography-tandem mass spectrometry (HPLC-MS/MS) method was developed and validated on AB Sciex 4500MD for the determination of chloroquine in human plasma. The samples were purified by protein precipitation (PP) and separated on a Kinetex Phenyl-Hexyl column (3.0 mm × 50 mm, 2.6 μm).

The validation results indicate that the method had excellent sensitivity and specificity. The linear range covered from 2.00 to 1000 ng/mL for chloroquine. Intra-run and inter-run precisions (in terms of %RSD) were all < 15% and the accuracies (in terms of %RE) were within the range of ±15%. In particular, the drug stability of conventional treatment and heating inactivation treatment (56 ℃, 30 min) was also investigated, and there was no significant difference in extraction recovery. Finally, the method was successfully applied to a clinical study of patients with COVID-19 after oral administration of chloroquine phosphate tablets.

The samples of 50 subjects in the text were measured in six batches. No significant problems, such as a shift in retention times, pressure ascend, and obvious interferences, were observed during the whole analysis procedure. The linear equation of this batch is Y=0.038X+0.00141 (R=0.9964). The accuracy of the quality control samples was 93.6%-105.4% with a variable coefficient of 5.4%.

**Table S1. Covariates to be evaluated on PK model parameters**

| Model parameters | Covariates to be evaluated |
| --- | --- |
| CL/F | Age; Sex; Weight; Body-mass Index; Alanine Aminotransferase; Aspartate Transaminase; Creatinine Clearance; Triglyceride; Combined use of Lopina Velitotavir; Combined use of Prazole |
| Q/F | / |
| V2/F | Age; Sex; Weight; Body-mass Index; Alanine Aminotransferase; Aspartate Transaminase; Creatinine Clearance; Combined use of Lopina Velitotavir; Combined use of Prazole |
| V3/F | / |
| ALAG | / |

Table S2. The summarization of population pharmacokinetic parameters from reference studies

| **Literature** | **N** | **CL(RSE%)** | **V2(RSE%)** | **Q(RSE%)** | **V3(RSE%)** | **KA(RSE%)** | **ALAG(RSE%)** | **IIVCL** | **IIVV2** | **IIVV3** | **IIVKA** |
| --- | --- | --- | --- | --- | --- | --- | --- | --- | --- | --- | --- |
| Celestino Obua 2007 | 86 | 2.84(5.4) | 378.4(20) | 3.29(11) | 2737(7.8) | 0.14(13) | / | 54.8(35) | 75.5(67) | / | / |
| Richard Höglund 2016 | 75 | 6.13(3.40) | 468(16) | 37.7(18.9) | 1600(5.21) | / | 0.733(43.1) | / | / | 20(61.6) | / |
| Qinying Zhao 2014 | 219 | 59.1(2.3) | 2870(4.3) | 61.4(11.3) | 1890(4.9) | 6.12(29.4) | 0.387(6.7) | 30.5(12.2) | 46.6(15.7) | / | / |
| Azrin N. Abd-Rahman 2020 | 24 | 54.6(5) | 2930(7) | 47.2(9) | 4700(7) | 0.943(21) | / | 29.0(36) | 42.3(27) | / | 81.4(24) |
| Sam Salman 2017 | 89 | 34.7(5) | 3380(8) | 23.6(13) | 5250(7) | 4.7(27) | 0.74(5) | 20(18) | 22(44) | / | 152(53) |
| **Meta-analysis** | **493** | **30.9(12.9)** | **1989.4(29.1)** | **34.1(32.5)** | **3162.1(14.4)** | **1.47(36.8)** | **0.59(27.1)** | **27.3(16.8)** | **38.6(19.1)** | **20(61.6)** | **85.3(22.3)** |
| **Degree of freedom** | **/** | / | / | / | / | / | / | **71.86** | **55.82** | **6.27** | **41.22** |

Table S3. The estimation of model parameter using the $PRIOR approach .

| **Parameter** | **Final estimate** | **%RSE** |
| --- | --- | --- |
| CL/F (L/h) | 29.2 | 12.7 |
| V2/F (L) | 3220 | 23.8 |
| Q/F (L/h) | 45.7 | 45.5 |
| V3/F (L) | 3770 | 21.6 |
| ka (h^-1^) | 1.53 | 2189.5 |
| ALAG1 (h) | 0.605 | 694.2 |
| ω^2^ for CL/F | 31.9 | 13.6 |
| ω^2^ for V2/F | 47.6 | 26.7 |
| ω^2^ for Q/F | 239.8 | 2591.3 |
| ω^2^ for V3/F | 45.7 | 28 |
| σ^2^ for aggregate data | 25.8 | 9.3 |

IIVs (ω^2^) and residual errors (σ^2^) are expressed as coefficients of variation (%).

Figure S1. Goodness of fit plots for the model using the $PRIOR approach.


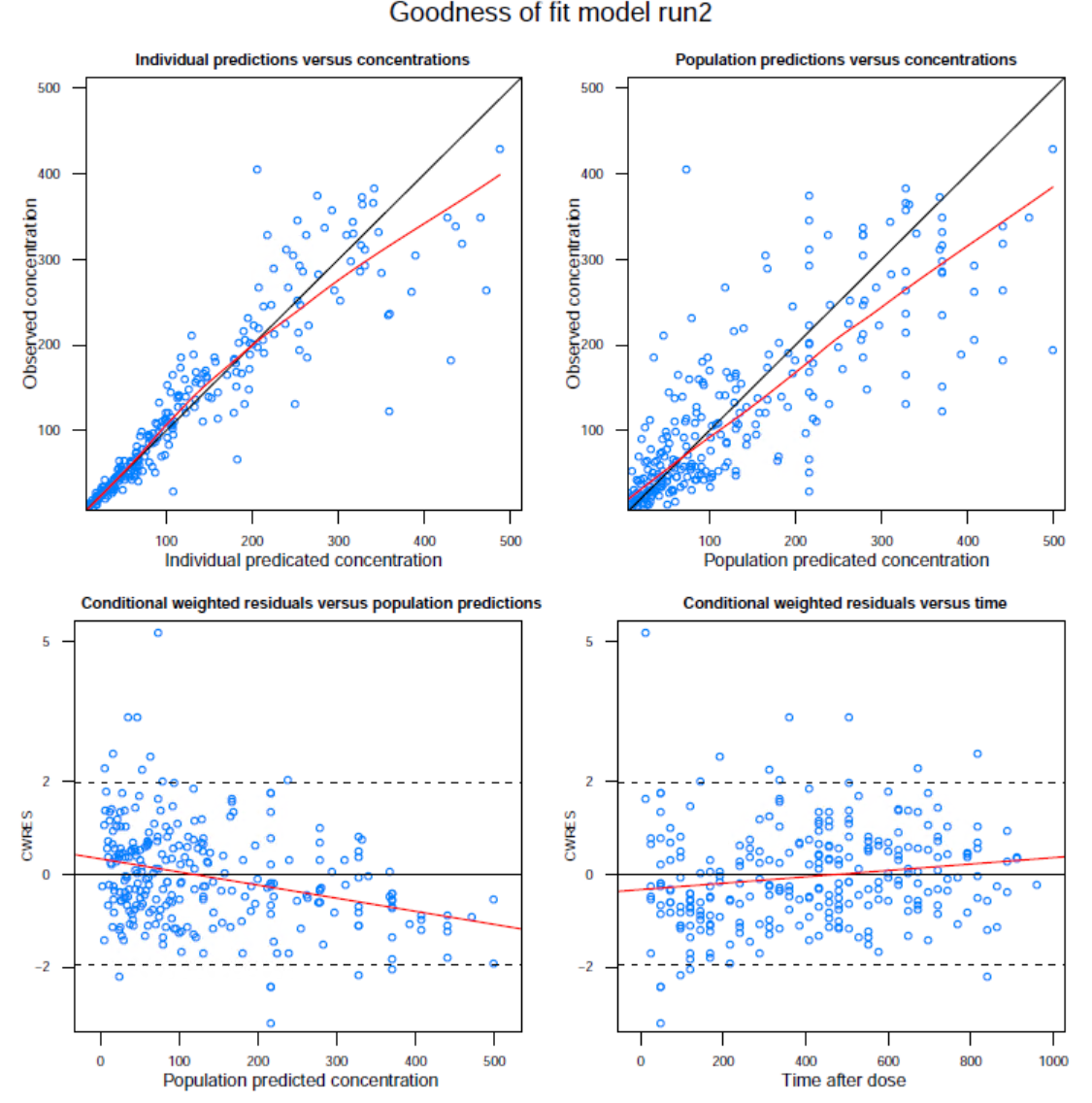

Supplement: Supplementary file 1 — (DOCX 452 kb) [file 228_2020_3032_MOESM1_ESM.docx]
